# Genetic Analysis of the Hsm3 Protein Function in Yeast *Saccharomyces cerevisiae* NuB4 Complex

**DOI:** 10.3390/genes12071083

**Published:** 2021-07-17

**Authors:** Tatiyana A. Evstyukhina, Elena A. Alekseeva, Dmitriy V. Fedorov, Vyacheslav T. Peshekhonov, Vladimir G. Korolev

**Affiliations:** 1 Laboratory of Eukaryotic Genetics, Department of Molecular and Radiation Biophysics, Petersburg Nuclear Physics Institute Named by B.P. Konstantinov of National Research Centre “Kurchatov Institute”, 188300 Gatchina, Russia; tat.evst@gmail.com (T.A.E.); dfv85@mail.ru (D.V.F.); pesh_vt@bk.ru (V.T.P.); korolev_vg@pnpi.nrcki.ru (V.G.K.); 2 Laboratory of Molecular Genetic and Recombination Technologies, Kurchatov Genome Center—Petersburg Nuclear Physics Institute, mkr. Orlova Roscha 1, Leningrad District, 188300 Gatchina, Russia

**Keywords:** yeast, *Saccharomyces cerevisiae*, NuB4 complex, *HSM3* gene, *HIF1* gene, UV-induced mutagenesis

## Abstract

In the nuclear compartment of yeast, NuB4 core complex consists of three proteins, Hat1, Hat2, and Hif1, and interacts with a number of other factors. In particular, it was shown that NuB4 complex physically interacts with Hsm3p. Early we demonstrated that the gene *HSM3* participates in the control of replicative and reparative spontaneous mutagenesis, and that *hsm3Δ* mutants increase the frequency of mutations induced by different mutagens. It was previously believed that the *HSM3* gene controlled only some minor repair processes in the cell, but later it was suggested that it had a chaperone function with its participation in proteasome assembly. In this work, we analyzed the properties of three *hsm3Δ*, *hif1Δ*, and *hat1Δ* mutants. The results obtained showed that the Hsm3 protein may be a functional subunit of NuB4 complex. It has been shown that *hsm3*- and *hif1*-dependent UV-induced mutagenesis is completely suppressed by inactivation of the Polη polymerase. We showed a significant role of Polη for *hsm3*-dependent mutagenesis at non-bipyrimidine sites (NBP sites). The efficiency of expression of *RNR* (RiboNucleotid Reducase) genes after UV irradiation in *hsm3Δ* and *hif1Δ* mutants was several times lower than in wild-type cells. Thus, we have presented evidence that significant increase in the dNTP levels suppress *hsm3*- and *hif1*-dependent mutagenesis and Polη is responsible for *hsm3*- and *hif1*-dependent mutagenesis.

## 1. Introduction

The chromatin of eukaryotic cells consists of nucleosome core particles containing a histone octamer core wrapped approximately two times with 147 bp of DNA. The histone core comprises a heterotetramer of two copies each of the histones H3 and H4 and two heterodimers of H2A and H2B [1]. Histones are highly charged basic proteins, which bind with chaperones that prevent them from interacting nonspecifically with other proteins and DNA and help regulate their proper deposition into nucleosomes [2].

The chromatin assembly of a genome imposes limitations on many cellular processes that require accessibility to chromosomal DNA. The posttranslational acetylation of the histone N-terminal tails has been shown to be an important mechanism by which cells regulate accessibility to chromatin [3]. The tail acetylation of newly synthesized H3 and H4 molecules is a transient modification. This modification changes both the charge and structure of lysine residues and is catalyzed by histone acetyltransferases.

Hat1 is the founding member of the class of enzymes known as type B histone acetyltransferases (HATs) [4]. Hat1p and Hat2p make up the core HAT1 complex [4]. Hat1p specifically acetylates lysine residues at positions 5 and 12 of the free histone H4 [5,6,7,8,9,10,11]. Genetic studies in yeast showed that the absence of histone H4 lysines 5 and 12 acetylation pattern had no effect on chromatin assembly and cell proliferation or viability [10,11,12,13,14]. Subsequent analyses indicated that Hat1 is actually a predominantly nuclear enzyme [15,16,17]. When in the nuclear compartment, Hat1 core complex interacts with a number of other factors. These interactions provide important clues to the functional role of Hat1.

Hat1 is most often represented in the nucleus of a yeast cell as a NuB4 complex that contains Hat1p, Hat2p, and Hif1p [16,17]. Hat2p possess histone chaperone activity and, therefore, are thought to mediate the interactions of these varied NuB4 complexes with histones [17,18,19,20,21]. Hif1p is a member of the N1 family of histone chaperones and specifically interacts with histones H3 and H4. Hif1p can participate in the deposition of histones onto DNA, suggesting that Hat1p may be directly involved in the chromatin assembly process [16]. It was shown that NuB4 complex physically interacts with Hsm3p [22,23,24]. It was shown earlier that the gene *HSM3* participates in the control of replicative and reparative spontaneous mutagenesis, and that *hsm3Δ* mutants increase the frequency of mutations induced by different mutagens [25,26,27,28,29,30,31]. It was previously believed that the *HSM3* gene controlled only some minor repair processes in the cell, but later it was suggested that it had a chaperone function with its participation in proteasome assembly [32,33,34]. This was confirmed quite recently: Takagi et al., 2012 [35] succeeded in establishing the spatial structure of the Hsm3 protein, as well as showing the interaction of this protein with the subunits of the proteasome complex. It means that that the product of this gene may have more than one functional domain. Analysis of various mutant alleles of the *HSM3* gene revealed that the C-terminal domain of the Hsm3 protein is responsible only for controlling induced and spontaneous mutagenesis and does not affect proteasome assembly [36].

To determine possible role of the *HSM3* and *HIF1* genes in HAT1 complex function, we conducted genetic analysis of properties of three *hsm3Δ*, *hif1Δ*, and *hat1Δ* mutants. Besides, our findings show that Polη is responsible for *hsm3*- and *hif1*-dependent mutagenesis.

## 2. Materials and Methods

Strains: *S. cerevisiae* strains used in this work are described in Table 1.

The *hat1Δ* (*CAY-2*) and *hif1Δ* (*CAY-3*) mutants were obtained from the previously described *LMG-3031* (*MATα ade2Δ-248 leu2-3,112 ura3-160,188 trp1*) strain [30] by gene replacement. A PCR-generated *natMX6* module and a PCR module containing the *ura4^+^* gene from *Schizosaccharomyces pombe* were amplified from pFLA6A-*natMX6* and pFLA6a-*ura4^+^* plasmids (Latypov) using *HAT1*_DelL: 5′-cgaatttattagatttctatgtatttctacttgaagttaggaatagatctttctggaattgttttcagcaaaattatgcttcgtacgctgcaggtcg-3′; *HAT1*_DelR: 5′-atatcatcgatgaattcgagcgacaacataacggcttcaacctttgcataagcttatattaactatagagacactttgatgatcatctcgatgatg-3′; and *HIF1*_DelL: 5′-atggggttacgtagtcgaaggatatagcagtgtctaaaaacttgcaagagcactcgtagcttcgtacgctgcaggtcg-3′; *HIF1*_DelR: 5′-atatcatcgatgaattcgagctccttatgaatagagaaaaatg actttttttagatgtgtaagtatgtcatttcagggatggtctgcttgctctttaaatattt-3′ deoxyoligonucleotides, respectively. The *LMG-3031* strain was transformed with those modules, and the transformants were selected on plates with YEPD containing 30 mg/L nourseothricin and on plates with selective media without uracil respectively. The double *hif1Δ hsm3Δ* (*CAY-9*) and *hsm3Δ hat1Δ* (*CAY-15*) mutants were constructed from previously described *5-LMG-3031* (*MATα ade2Δ-248 leu2-3,112 ura3-160,188 trp1 hsm3::Kan ^R^*) strain [30], as described above. The double *hif1Δ hat1Δ* (*CAY-11*) and the triple *hif1Δ hsm3Δ hat1Δ* (*CAY-10*) mutants were obtained by replacement of *HAT1* gene in *hif1Δ* (*CAY-3*) and *hif1Δ hsm3Δ* (*CAY-9*) strains, according to the same procedure. All mutants were PCR-verified.

The double *hat1Δ rad1Δ* (*CAY-6*) mutant was constructed by transforming the *hat1Δ* (*CAY-2*) strain with pJH552 (R. Keil) plasmid digested by SalI. The double *hat1Δ rad2Δ* (*CAY-7*) and *hif1Δ rad2Δ* (*CAY-12*) mutants were obtained by transforming *hat1Δ* (*CAY-2*) and *hif1Δ* (*CAY-3*) strains with pWS521 (W. Siede) plasmid digested by SalI. The double *hat1 Δrad52Δ* and *hif1Δ rad52Δ*were produced by transforming *hat1Δ* and *hif1Δ* strains with pJH183 and pJH181 plasmids, respectively, digested by BamHI. All transformants were selected on plates with proper selective media.

In comparative experiments previously described, LMG-351 (*MATα ade2Δ-248 leu2-3,112 ura3-160,188 trp1 rad1::LEU2*) [29], LMG-352 (*MATα ade2Δ-248 leu2-3,112 ura3-160,188 trp1 rad2::TRP1*) [37], 10-CAY-3031 (*MATα ade2Δ-248 leu2-3,112 ura3-160,188 trp1 rad52::URA3*) [38], and 13-CAY-5-3031 (*MATα ade2Δ-248 leu2-3,112 ura3-160,188 trp1 hsm3::KanR rad52::URA3*) [38] strains were used.

**Media:** Standard yeast media of complete and minimal composition were used in the work [39]. In some experiments, a liquid YPD was used without the addition of agar. When working with auxotrophic mutants, metabolites required for growth were added to the minimal medium at a rate of 20 mg/L for amino acids and 3 mg/L for nitrogen bases. As a selective medium for accounting for the frequency of canavanine resistance mutations, a minimal medium was used with the addition of a liquid YPD in an amount of 10 mL/L and required for the growth of amino acids and nitrogenous bases. Depending on the strains used, canavanine concentrations were up to 80 mg/L. Taking into account the frequency of induced mutations at five loci, YPD with an alcohol instead of glucose was used, the composition of which was described earlier [37].

**Sensitivity against UV irradiation:** Cell killing tests were performed on plates by growing overnight a culture of the respective strain in liquid YPD at 30 °C. Cells were washed and resuspended in water at a density of 1 × 10^7^ cell/mL. Cells were irradiated with a UV lamp BUV-30 (UV-C range). Aliquots were withdrawn at different times, diluted, and plated onto YPD plates to determine the number of survivors.

**Mutation frequency:** Mutation tests were performed on plates by growing overnight a culture of the respective strain in liquid YPD at 30 °C. Cells were washed and resuspended in water at a density of 1 × 10^7^ cell/mL. Cells were irradiated with a UV lamp BUF-30. Aliquots were withdrawn at different times, diluted, and plated onto YPD plates to determine the number of survivors. To determine the mutation frequency, undiluted aliquots were plated onto a medium YPD with an alcohol instead of glucose, the composition of which was described earlier [37].

**Mutation rates:** Mutation rates were determined according to the methods: fluctuation test [40] and ordered seeding [41]. The first method allows the determination of the rate of spontaneous yeast cell mutations in the process of fast growth on complete medium. After incubation for three days, 12 separate colonies were scored, and each colony was suspended in 1 mL of water and plated on selective medium with canavanine, at a concentration that rules out the possibility of growth of canavanine-sensitive cells. When estimating the number of plated cells, we diluted suspensions and plated them on complete medium. After incubation for three or four days, the number of canavanine-resistant colonies and the total number of cells on the plate were counted. The occurrence frequency of spontaneous mutations was estimated using the special formula [32].

Using the method of perfect order plating, one can register the frequency of spontaneous mutations arising in the process of slow growth on selective medium containing lower concentrations of canavanine in which cells are grown over 8 to 10 divisions. Cells were incubated in 2 mL of complete liquid medium for 2 days; next, 1 mL of grown culture was diluted in 5 mL of water. A special replicator having 150 appliances was embedded into the suspension, and drops were placed onto plates with selective medium. After a14-day incubation, the number of canavanine-resistant colonies and the total number of cells grown in 150 spots was counted. The number of grown non-mutant cells was determined after washing away cells from individual reprints on which no canavanine-resistant colony was visualized. The rate of mutation was determined by dividing the number of canavanine-resistant colonies by the number of cells in all reprints [33].

In total, five replicates of the experiment are shown on the graphs and in the tables, and the mean values with 95% confidence intervals are given.

**Real-time PCR:** For conducting Real-time PCR was used on a CFX96 RT-PCR Detection system (Bio-Rad, Watford, UK). The reactions were carried out in 25 µL volumes consisting of 10 µL 2,5-fold reaction mixture for RT-PCR in the presence SYBR Green I dye and Rox reference dye (Syntol, Moscow, Russia), 14.1 µL water, 0.7 µL of cDNA and 0.1 (2 mM) respective primers (primers for gene *RNR3*: For*RNR3* 5′-ACACCTTTCATGGTTTATAAG-3′ and Rev*RNR3* 5′-CGACGATTTCACAACATAA-3′; for gene *ACT1*: For*ACT1* 5′-GAAGGTCAAGATCATTGC-3′ and Rev*ACT1* 5′- GTTGGAAGGTAGTCAAAG-3′).

PCR cycling conditions were as follows: 1 cycle of 5 min at 95 °C, followed by 39 cycles of 15 s at 95 °C and 20 s at 52 °C. Melting curve analysis was 5 s incremental increases of 1 °C from 55 °C to 95 °C.

Control reactions with primer and template free reaction mixtures were included. Two biological and three technical replicates were performed for each sample. The results were processed using the CFX Manager program (Bio-Rad, Watford, UK).

**Statistical analysis:** Experimental data are shown as the means standard deviations from at least three biological replicates, and statistical differences were determined by the Student’s *t*-test. Significance was determined at the level of *p* < 0.05.

## 3. Results

The physical interaction between the products of the genes *HAT1*, *HIF1*, and *HSM3* has been demonstrated in previous studies [22,23,24]. We suppose that this interaction plays a functional role and Hsm3 protein is a subunit of NuB4 complex. In this work, we conducted a comparative study of the genetic properties of *hif1Δ* and *hsm3Δ* mutations and their interaction with a deletion mutation in the *HAT1* gene, which codes for the catalytic subunit of the complex.

***HSM3*and*****HIF1**:* It has been shown that the mutants for the *HAT1* gene affect the repair processes in yeast [42]. Therefore, we first tested spontaneous mortality of the single *hif1Δ* and *hsm3Δ* mutants. The percentage of lethal clones was assessed after a day of incubation in solid complete medium. Clones with fewer than 16 cells were considered to be lethal. In the test for spontaneous mortality, both mutants showed the same pattern. These mutants did not change significantly the rate of spontaneous death of cells compared to the wild-type strain (Table 2). According to our research and the data presented in work [38], in the double *hif1Δ rad52Δ* and *hsm3Δ rad52Δ* mutants, *hif1Δ* and *hsm3Δ* mutants were equally epistatized to recombination-deficient mutants. These data support the hypothesis advanced earlier that *hsm3Δ* mutation (maybe *hif1Δ*) leads to the destabilization of the D-loop during the post-replicative repair [38,43] and thus reduces the load on the path of the recombination repair, which is blocked in *rad52Δ* mutant.

**Figure 1:**
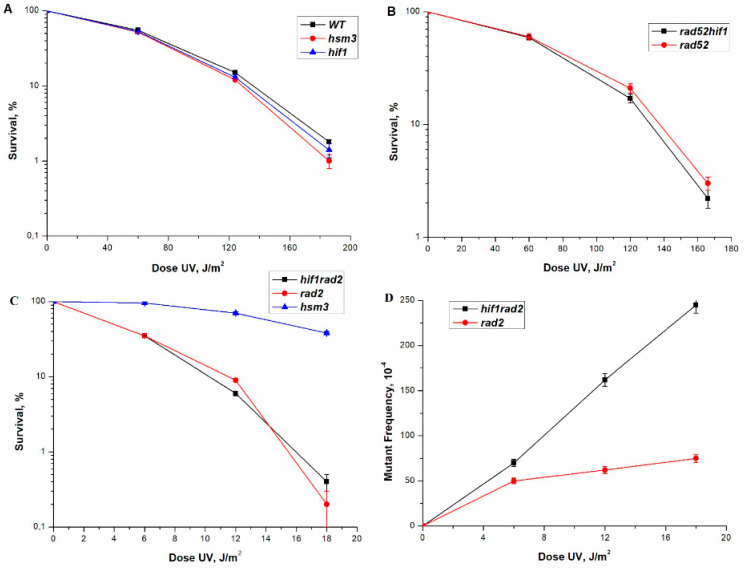
Sensitivity and mutagenesis in various mutant strains at different UV doses. Cells were irradiated at the indicated dose, the viable titer was determined, and the percentage of survivals was calculated. The mutation frequencies were determined, as a ratio of the number of white colonies to the number of all colonies grown in a cup with complete medium. The mean ± SEM values obtained from four independent experiments are plotted. (**A**) UV-induced sensitivity in strain wild-type (LMG-3031) and mutant strains *hsm3Δ* (5-LGM-3031), *hif1Δ* (CAY-3); (**B**) UV-induced sensitivity in mutant strains *rad52Δ hif1Δ (CAY-13)*, *rad52Δ* (10-CAY-3031); (**C**) UV-induced sensitivity mutagenesis in mutant strains *hif1Δ rad2Δ* (CAY-12), *rad2Δ* (LGM-352), *hsm3Δ* (5-LGM-3031); (**D**) UV-induced mutagenesis in mutant strains *hif1Δ rad2Δ* (CAY-12), *rad2Δ* (LGM-352).

**Figure 2:**
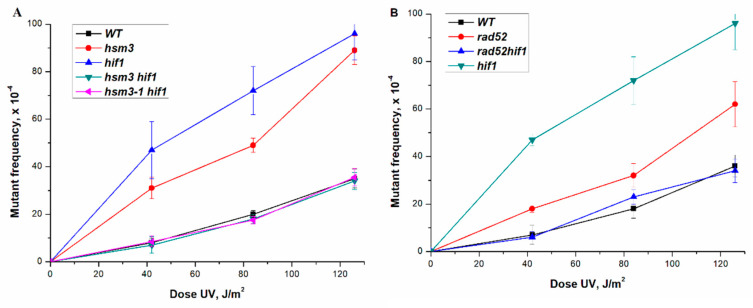
Dependence of the frequency of induced mutagenesis on the dose of UV rays. The mean ± SEM values obtained from 4 independent experiments are plotted. (**A**) Frequencies of UV-induced mutations in strain wild-type (LMG-3031) and mutant strains *hsm3Δ* (5-LGM-3031), *hif1Δ* (CAY-3), *hsm3Δ hif1Δ* (CAY-9), *hsm3-1* *hif1Δ* (TAE155); (**B**) Frequencies of UV-induced mutations in wild-type (LMG-3031) and mutant strains *rad52Δ* (10-CAY-3031), *rad52Δ hif1Δ* (CAY-13), *hif1Δ* (CAY-3).

To further test the survival of *hsm3Δ* and *hif1Δ* mutants, we performed experiments with UV light. We found that both single mutants showed the same sensitivity to UV as a wild-type strain (Figure 1A). The double *hsm3Δ hif1Δ* mutant practically did not differ in this parameter from single mutants (Figure 2A). Thus, mutations in the *HIF1* and *HSM3* genes have no significant effect on the survival of yeast cells after UV irradiation.

In order to study the role *hsm3Δ* and *hif1Δ* mutants in the DNA damage response, we were constructed double mutant strains with genes from the major DNA repair pathways. Nucleotide excision repair-deficient strain, *rad2Δ*, was used for *RAD3* epistasis group, while *rad52Δ* was chosen for the recombination repair epistasis group. The *hif1Δ* mutation, like the *hsm3Δ* mutation, in combination with *rad2Δ* did not lead to a change in the UV resistance of the double mutant *hif1Δ rad2Δ* cells compared to the cells of a single *rad2Δ* mutant (Figure 1C,D). Based on the data obtained, it can be concluded that *hsm3Δ* and *hif1Δ* mutations do not affect NER. *rad54Δ* and *rad52Δ* mutants are weakly sensitive to UV, which corresponds to the previously obtained data [38]. Earlier, we have shown that *hsm3Δ* mutation does not change the UV sensitivity of *rad52Δ* mutant [38]. Double *hif1Δ rad52Δ* mutant had the same sensitivity as single radiosensitive mutant (Figure 1B). Taken together, *hsm3Δ* and *hif1Δ* mutations did not affect the radiosensitivity of yeast cells.

It has been shown that the *HSM3* gene participates in the control of replicative and reparative spontaneous mutagenesis [29]. Therefore, we set out to compare the spontaneous mutagenesis of the *hif1Δ* mutant to that of the *hsm3Δ* mutant. When studying the rate of occurrence of spontaneous mutations, we used two different methods: the fluctuation test (the “Coulson-Lee median” method), which that allows to determine level of spontaneous replication mutagenesis, and the method of ordered seeding, which can be judged on the level of reparative mutagenesis. In both cases, the frequency of mutations in the *CAN1* gene was calculated. From the data in Table 3 it is seen that that the presence of *hsm3Δ* and *hif1Δ* mutations in cells leads to a slight change in the rate of replicative spontaneous mutations compared to wild-type cells. In the ordered seeding test, both mutations led to a sharp increase in the rate of spontaneous mutagenesis. *hsm3Δ* and *hif1Δ* mutations increased the rate of spontaneous reparative mutagenesis in this test by approximately 18 times. These results support the notion that *hsm3Δ* and *hif1Δ* have the same phenotype.

Earlier, we studied in detail the effect of *hsm3Δ* mutation on UV-induced mutagenesis [29]. As shown previously, *hsm3Δ* mutation significantly increased the induced mutagenesis when exposed to various mutagenic factors. As shown in Figure 2A, the *hif1Δ* mutation, like *hsm3Δ* mutation, significantly increased the frequency of UV-induced mutagenesis. Unexpectedly, the double *hsm3Δ hif1Δ* mutant showed the level of UV-induced mutagenesis peculiar to wild-type strain, as well as a double mutant *hsm3-1 hif1Δ* mutant with a point mutation of the C-terminal domain of the Hsm3p. (Figure 2A). Hence the loss of both the Hif1p and Hsm3p results in increased UV-induced mutagenesis; inactivation of both proteins leads to a phenotype corresponding to the wild-type cells.

In all previous experiments, we used an unsynchronized cell culture. As it is known, in such a culture there is a mixture of cells in the G1, S, and G2 phases. The repair of UV-induced damage occurs by the mechanism of the nucleotide excision repair (NER) at all three stages of the cell cycle. In NER-defective strains, damage repair will be performed in the S and G2 phases by the process of postreplicative and recombination repairs. To assess the effect of mutations in the genes encoding the subunits of the HAT1 complex, we have disrupted the key gene that provides NER in the single mutants mentioned above. *hsm3Δ* mutation significantly increases the mutagenesis of *rad2Δ* mutant with broken nucleotide excision repair [37]. We tested how the mutation in the *HIF1* gene, which codes for another subunit of the NuB4 complex, affects UV mutagenesis. As shown in Figure 1B, the interaction of *rad2Δ* and *hif1Δ* mutations has a synergistic character, as in the case of the interaction of *hsm3Δ* and *rad2Δ* mutations. Earlier, we had shown that *rad52Δ* mutation suppressed UV-induced mutagenesis in *hsm3Δ* significantly [38]. Therefore, we wished now to compare the UV-induced mutagenesis of *hif1Δ* mutant with that of *rad52Δ* mutant. *rad52Δ* mutation suppressed the UV-induced mutagenesis of *hif1Δ* mutant to wild-type strain level (Figure 2B). In summary, *hsm3Δ* mutation may be considered a phenocopy of *hif1Δ* mutation.

However, the *rad52Δ* mutation suppresses *hsm3*- and *hif1*-dependent UV-induced mutagenesis in different ways. In the *hif1Δ* mutant, the *rad52Δ* mutation suppresses the frequency of UV-induced mutations to the wild-type level, while in the *hsm3Δ* mutant the *rad52Δ* mutation slightly reduces the frequency of UV-induced mutations [38]. There was also a difference in the frequency of UV-induced mutagenesis in single mutants *hsm3Δ* and *hif1Δ* (Figure 2A).

**Figure 3:**
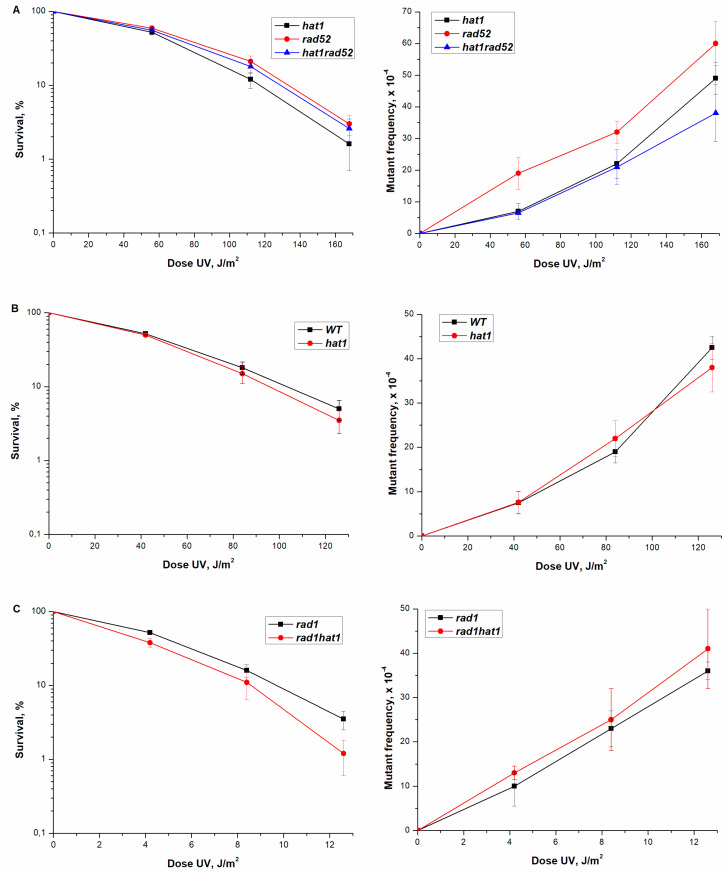
Sensitivity and mutagenesis in different strains after UV irradiation at different doses. The viable titer was determined, and the percentage of survivals was calculated. The mean ± SEM values obtained from 4 independent experiments are plotted. (**A**) UV-sensitivity and mutation frequency in mutant strains *hat1Δ* (CAY-2), *rad52Δ* (10-CAY-3031), *hat1Δ rad52Δ* (CAY-4); (**B**) UV-sensitivity and mutation frequency in strain wild-type (LMG-3031) and mutant strain *hat1Δ* (CAY-2); (**C**) UV-sensitivity and mutation frequency in mutant strains *rad1Δ* (LMG-351), *rad1Δ hat1Δ* (CAY-6).

***HAT1*:** Hat1p is the catalytic subunit of HAT complex. Therefore, we first tested for the genetic properties of *hat1Δ* mutant. The spontaneous mortality of single *hat1Δ* and double *hat1Δ rad52Δ* mutants compared to that of the wild-type strain. *rad52Δ* and *hat1Δ* single mutations increased the percentage of lethal clones to 10.1 ± 3.15 and 11.4 ± 3.97% respectively (Table 2). Double mutants *hat1Δ rad52Δ* did not change the frequency of spontaneous cell death compared to single mutants (Table 2). Thus, there is an epistatic interaction of *hat1Δ* mutation with mutations that block the recombination repair. This conclusion is supported by data comparing the UV sensitivity of a single mutant *hat1Δ* with the UV sensitivity of double mutants *hat1Δ rad52Δ*. Both strains, studied, showed the same UV sensitivity (Figure 3A). Thus, in *hat1Δ* mutant, the recombination repair pathway of spontaneous and UV-induced lesions is destroyed.

Next, we tested the UV sensitivity of wild-type strain versus a single *hat1Δ* mutant. A single mutant showed a sensitivity to UV comparable to the sensitivity of a wild-type strain (Figure 3B). Therefore, mutant *hat1Δ* does not affect NER. This conclusion is confirmed by the results obtained for the interaction of *hat1Δ* mutation and *rad1Δ,* which blocks NER. The double *rad1Δ hat1Δ* mutant showed UV sensitivity equal to a single *rad1Δ* mutant (Figure 3C).

Early the genetic effects of mutations in *HAT1* gene have not been adequately studied. That is why we studied the effect of the *hat1Δ* mutation on the mutation process. From the data of Table 3, it can be seen that the presence of *hat1Δ* mutation results in a slight change in the rate of replicative spontaneous mutations compared to wild-type cells. However, in the ordered seeding test, *hat1Δ* mutation leads to a sharp increase in the rate of spontaneous mutagenesis (Table 3).

The frequency of direct mutations, in the loci of *ADE4-ADE*8, induced by UV rays in a wild-type strain and a single *hat1Δ* mutant was measured. The data presented in Figure 3B suggests that *hat1Δ* mutation does not affect the frequency of UV-induced mutagenesis.

As can be seen from Figure 3C single *rad1Δ* mutant and the double *hat1Δ rad1Δ* mutant have approximately the same level of mutagenesis. Single *rad52Δ* mutant shows greater UV-mutability compared to wild-type strain [38]. Double *hat1Δ rad52Δ* mutant at low doses showed the level of mutagenesis characteristic of a single *hat1Δ* mutant (Figure 3A). Thus, in this test, *hat1Δ* mutation at low doses epistatizes to *rad52Δ* mutation.

It is known that the Hat1 subunit is catalytic in the HAT1 complex. In connection to this, we studied the epistatic interaction of *hat1Δ* mutation with mutations in genes, coding for other subunits of the complex. As shown in Table 3, the spontaneous mutation rates in double *hat1Δ hsm3Δ* and *hat1Δ hif1Δ* mutants do not differ from the spontaneous mutation rate in single hat1Δ mutant. These data corroborate previous results that *hat1Δ* epistatized to both single *hsm3Δ* and *hif1Δ* mutants.

**Figure 4:**
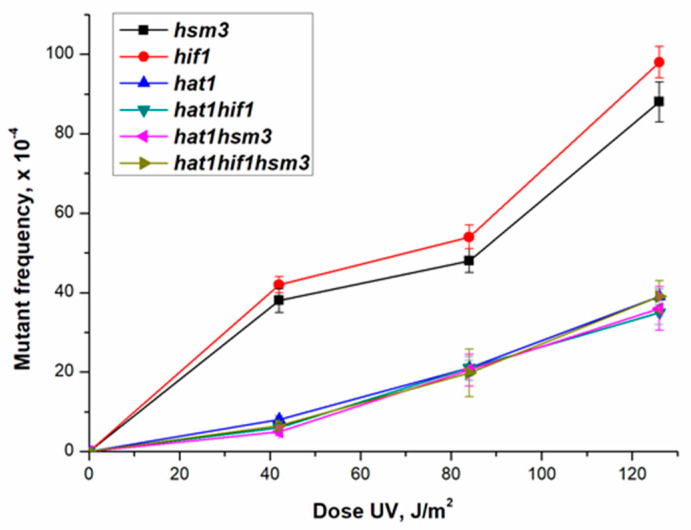
UV-induced mutagenesis in mutant strains *hsm3Δ* (5-LGM-3031), *hif1Δ* (CAY-3), *hat1Δ* (CAY-2), *hat1Δ hif1Δ* (CAY-11), *hat1Δ hsm3Δ* (CAY-15), and *hat1Δ hif1Δ hsm3Δ* (CAY-10) at different UV doses.

Figure 4 shows the dependence of the mutagenesis frequency on the dose of UV rays for single *hat1Δ*, *hsm3Δ* and *hif1Δ* mutants, double *hat1Δ hif1Δ*, *hat1Δ hsm3Δ*, *hsm3Δ hif1Δ* mutants and triple *hat1Δ hsm3Δ hif1Δ* mutant. As can be seen from this figure, *hat1Δ* mutation epistatizes to all studied mutations.

Taken together, our results argue that *hsm3Δ* and *hif1Δ* mutants have the same phenotypes, and that *hat1Δ* mutation epistatizes to these mutations in all the used tests. Thus, Hsm3 protein may be a new subunit of NuB4 complex.

**Figure 5:**
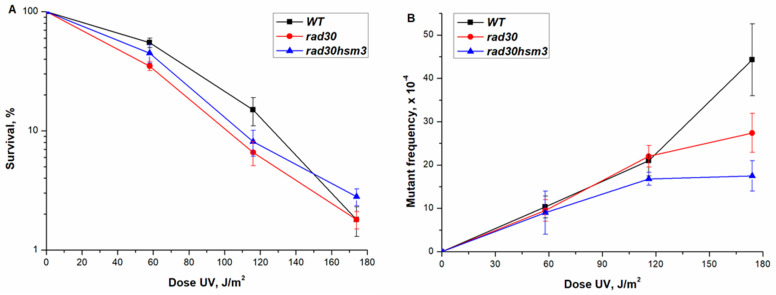
Sensitivity and mutagenesis in different strains after UV irradiation at different doses. The viable titer was determined, and the percentage of survivals was calculated. The mean ± SEM values obtained from four independent experiments are plotted. (**A**) UV-induced sensitivity in strain wild-type (LMG-3031) and mutant strains *rad30Δ* (TAE-153), *rad30Δ hsm3Δ* (TAE-152); (**B**) Frequencies of UV-induced mutations in wild-type (LMG-3031) and mutant strains r*ad30Δ* (TAE-153), *rad30Δ hsm3Δ* (TAE-152).

**Interaction between *RAD30* and *HSM3:*** Evidence has been obtained showing that *HSM3* and *HIM1* genes play a role in stabilizing the D-loops [42,44]. Earlier, we showed that after the destruction of the D-loop in the *him1* mutant, Polη fills the remaining gap (44). Based on these data, we hypothesized that the cause of *hsm3*-mediated UV-induced mutagenesis as well as *him1*-dependent UV-mutagenesis is the replacement of Polδ with highly erroneous Polη. To test this assumption, we have studied UV-induced mutagenesis in *rad30* and *rad30 hsm3* mutants. *rad30Δ* single mutant showed UV-induced mutagenesis as the wild-type strain (Figure 5). At low doses, double mutant showed the same level of UV-induced mutagenesis as single *rad30Δ* mutant. However, at high doses, UV-induced mutagenesis in the double mutant was noticeably lower than in the single *rad30Δ*. At the same time, double mutant showed high UV resistance than the single *rad30Δ*. We observed the same tendency in the case of *him1Δ rad30Δ* mutant [44]. Thus, we can conclude that, in during PRR the Polη in *hsm3Δ* mutant carries out reparative synthesis in unfilled gaps.

UV-induced mutations at bipyrimidine sites during TLS (TransLesion Synthesis) arise as a result of bypassing DNA damage. Mutations at non-bipyrimidine sites frequently occur on an intact template during polymerase Polη repair synthesis. It is known that the *CAN1* gene sequence contains 77% bipyrimidine sites and 23% non-bipyrimidine sites (NBP) [45]. In order to find out the ratio of UV-induced mutations in non- and bipyrimidine sites in mutant *rad30*, mutation spectra were determined at the *CAN1* locus in *hsm3Δ* strain. We used the same scheme and experimental conditions as in the work with *him1Δ* mutant [44]. 100 *can ^R^* mutants were isolated after UV irradiation at a dose 84 J/m^2^.

The spectrum of mutations obtained by us in *hsm3Δ* mutant practically obtained in the work [44]. The UV-induced spectra generated in *hsm3Δ* background does not differ from the mutation spectra in *him1Δ* mutant and was characterized by a predominance of single base substitutions (Table 4).

In NBP sites, the frequency of UV-induction mutations also practically does not differ between *hsm3Δ* (21 × 10^−5^) and *him1Δ* (19 × 10^−5^) and was significantly different from *him1Δ rad30Δ* strains (1 × 10^−5^). Taken together, data obtained suggests a key role for Polη in *hsm3*-dependent mutagenesis, especially at NBP sites.

**Figure 6:**
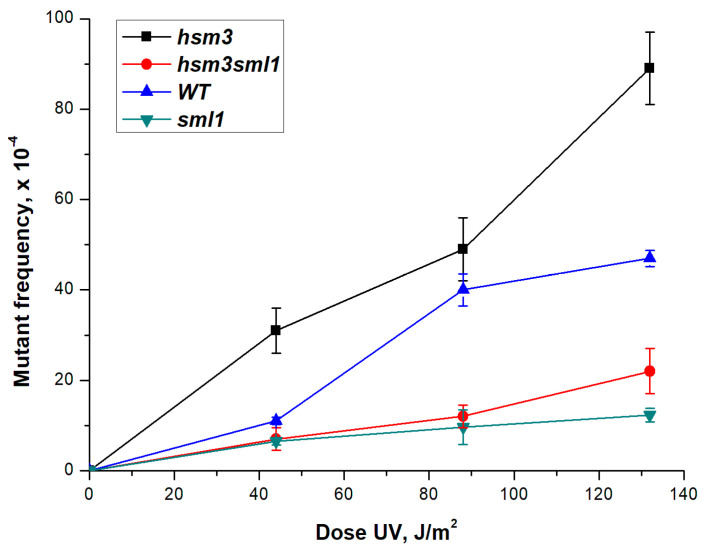
UV-induced mutagenesis in strain wild-type (LMG-3031) and mutant strains *hsm3Δ* (5-LGM-3031), *hsm3Δ sml1Δ* (TAE-151), *sml1Δ* (6- DVF-3031) at different UV doses. The mutation frequencies were determined, as a ratio of the number of white colonies to the number of all colonies grown in a cup with complete medium. The mean ± SEM values obtained from 4 independent experiments are plotted.

***RNR3* expression in *hsm3, hif1,* and *hat1* mutants:** Earlier, we showed that the reason for the change of polymerases in *him1Δ* mutant is a significant decrease in the level of dNTPs in mutant cells [44]. dNTP levels show a three- to five-fold increase in response to DNA damage relative to a normal S-phase, through the check-point-dependent induction of *RNR* genes, the allosteric regulation of RNR activity and the degradation of the Rnr1 inhibitor Sml1 [46,47,48]. To determine the role of the dNTPs pool in *hsm3Δ*-dependent mutagenesis, we deleted the *SML1* gene in wild-type and *hsm3Δ* mutant strains. *SML1* gene encodes a specific suppressor of the *RNR1* gene (*RNR3* homologue). Deletion of the *SML1* gene lowers the level of UV-induced mutagenesis in comparison with the wild-type strain (Figure 6). Thus, high level of dNTP pool suppresses *hsm3*-dependent mutagenesis.

**Figure 7:**
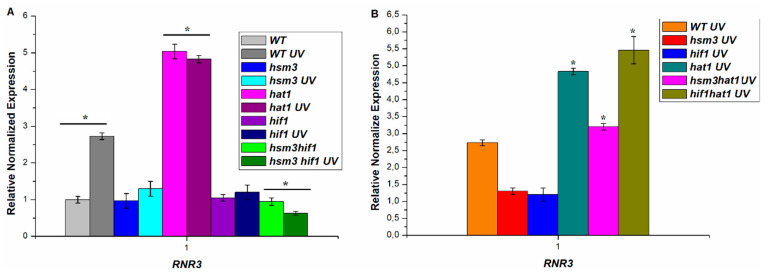
Relative normalized expression of the *RNR3* gene, before and after irradiation with UV light of the corresponding strains (after UV irradiation, the cells were kept for two hours at 30 °C in a thermostat for induction), UV dose at 252 J/m^2^; * *p* < 0.05, Student’s t test. (**A**) Relative normalized expression of the *RNR3* gene, before and after irradiation with UV light; (**B**) Relative normalized expression of the *RNR3* gene, after irradiation with UV light.

To test these results that dNTP concentration regulates UV-induced mutagenesis, we studied the expression of *RNR3* gene in the *hsm3Δ* mutant after UV irradiation. We measured the mRNA *RNR3* gene levels in the wild type, *hsm3Δ*, *hif1Δ*, *hsm3Δ hif1Δ*, *hsm3Δ hat1Δ*, *hif1Δ hat1Δ,* and *hat1Δ* mutant cells 2 h after irradiated with UV light. The mRNA level in wild-type cells increased almost three times, while in mutant cells the increase did not reach 30% (Figure 7). Thus, *hsm3Δ* and *hif1Δ* mutations suppress the efficiency of the induction expression of *RNR* genes after UV irradiation. The consequence of the suppression of the expression of *RNR* genes will be a decrease in the dNTP concentration. Taken together, the results confirm the hypothesis that that suppression of UV-induced expression of *RNR* genes stimulate Polη recruitment to fill the gaps. Polη is highly erroneous polymerase and this is the cause of the increased UV-induced mutagenesis in *hsm3Δ* and *hif1Δ* mutants.

As can be seen in Figure 7A, the level of expression of the *RNR3* gene in *hif1Δ hsm3Δ* double mutant drops below the level of expression of this gene in wild type cells without irradiating. This result allows us to conclude that inactivation of both accessory subunits NuB4 complex Hsm3 and Hif1 completely suppresses UV-induced expression of RNR complex genes. It is possible that such a sharp decrease in the expression of the *RNR* genes was the cause of the suppression of mutagenesis in the double mutant. To test this assumption, we decided to use the *dun1Δ* mutant.

**Figure 8:**
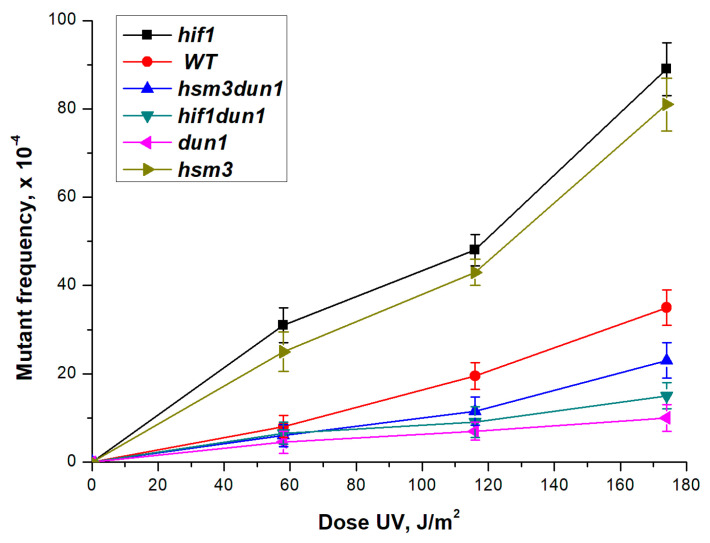
UV-induced mutagenesis in mutant strains *hif1Δ (CAY-3*), *hsm3Δ dun1Δ (DVF-15)*, *hif1Δ dun1Δ (TAE-154*), *dun1Δ (DVF-16)*, *hsm3Δ (5-LGM-3031),* and strain wild-type *(LMG-3031)* at different UV doses. The mutation frequencies were determined, as a ratio of the number of white colonies to the number of all colonies grown in a cup with complete medium. The mean ± SEM values obtained from four independent experiments are plotted.

In *dun1* mutant cells, there is no increase in the expression of *RNR* genes after DNA damage [45,49]. We deleted the *DUN1* gene in strains of wild-type, *hif1Δ* and *hsm3Δ* mutants. *dun1Δ* mutation significantly increases the sensitivity of yeast cells to UV radiation (Figure 8). At the same time, the double *dun1Δ hsm3Δ* and *dun1Δ hif1Δ* mutants did not differ from the single *dun1Δ* mutant under these conditions. Single *dun1Δ* mutant decreases the frequency of UV-induced mutagenesis compared to a wild-type strain (Figure 8). Simultaneously, the double *dun1Δ hsm3Δ* and *dun1Δ hif1Δ* mutants does not differ practically from the single *dun1Δ* mutant according to the frequency of UV-induced mutagenesis. Taken together, these results show that a sharp decrease in the dNTP concentration suppress *hsm3*- and *hif1*-dependent mutagenesis.

As seen from Figure 3B, *hat1Δ* mutation does not significantly affect the frequency of UV-induced mutagenesis. This is surprising, since mutations in the genes encoding the two subunits of the NuB4 complex increase the frequency of UV-induced mutagenesis. To examine the role *hat1Δ* mutation in *RNR3* regulation, we measured the expression level of the *RNR3* gene in *hat1* mutant before and after UV irradiation and the double *hsm3Δ hat1Δ*, *hif1Δ*, *hat1Δ* mutants after UV irradiation. Figure 7A shows that *hat1Δ* mutation significantly increases the expression of the RNR complex genes both before and after irradiation. We have shown that *hat1Δ* mutation epistats to *hsm3Δ* and *hif1Δ* mutations, also increases the expression of the RNR complex genes after UV irradiation, as in the single *hat1Δ* mutant (Figure 7B). This result explains the absence of increased mutagenesis in *hat1Δ* mutant and once again proves the key role of a decreased level of dNTP concentration in *hsm3*- and *hif1*-specific mutagenesis.

## 4. Discussion

Histone chaperone proteins have key roles in eukaryotic chromatin dynamics [50,51]. These proteins have been implicated in a wide range of processes including buffering of soluble H3-H4-complex [52], mediating H4 acetylation in the context of HAT1-complex [16,17]. In spite of these roles, histone chaperone proteins in various chromatin related processes, underlying mechanistic details are unclear. Here, we have shown that Hsm3 protein may be a new subunit of NuB4 complex.

Earlier in our laboratory, extensive research was carried out on the genetic properties of *hsm3Δ* mutation [25,26,27,28,29,30,31]. We have shown that proteins Mms2, Xrs2, Srs2, Mph1, Mms4, involved in the error-free branch of the PRR, have a crucial function in *hsm3*-dependent UV mutagenesis [38,43]. These results strongly suggest that the *HSM3* gene is involved in the error-free branch of damage bypass.

Several studies previously established that Hsm3 physically interacts with Hat2, Hif1, and histone H4 [22]. In this regard, we carried out a comparative study of the genetic properties of *hsm3Δ* and *hif1Δ* mutations. In all tests carried out, both mutations showed the same properties. *hsm3Δ* and *hif1Δ* mutations did not affect the radiosensitivity of yeast cells, equally increased the frequency of UV-induced mutagenesis and the rate of spontaneous reparative mutagenesis. Both mutations do not affect spontaneous cell death, but they suppress spontaneous death of recombination-deficient mutants lowering the level of spontaneous death in double mutants to the level characteristic of single *hsm3Δ* and *hif1Δ* mutants (Table 2). In the same time *rad52Δ* mutation suppressed the *hif1*- and *hsm3*-specific UV-induced mutagenesis. The interaction of *rad2Δ* and *hif1Δ* and *hsm3Δ* mutations in UV-induced mutagenesis has a synergistic character. The data obtained allowed us to conclude that *hsm3Δ* mutation may be considered a phenocopy of *hif1Δ* mutation.

To date, there is no genetic evidence for the participation of Hsm3 protein in the NuB4 complex in the literature. The data obtained during the experiments on the study of UV-induced mutagenesis in *hat1Δ*, *hif1Δ*, and *hsm3Δ* mutants showed that such participation is possible (Figure 4). It can be seen from Figure 4 that the level of mutagenesis in *hif1Δ* and *hsm3Δ* mutants is the same and significantly exceeds that of *hat1Δ* mutant. In the double mutants, the mutation of *hat1Δ* epistatizes to both *hif1Δ* and *hsm3Δ* mutations. This conclusion is supported by the data of the epistatic analysis of *hat1Δ, hsm3Δ, hif1Δ*, and *rad2Δ* mutations, as well as the data on the rate of spontaneous mutagenesis (Table 3). In all tests, *hat1Δ* mutation epistatized to *hif1Δ* and *hsm3Δ* mutations, thus confirming the same result.

When using the Coulson method, the cells were grown on a rich medium; the generation time for such growth was short and the amount of spontaneous damage that generated mutations during replication was relatively small. That is, the frequency of spontaneous errors of DNA polymerases on an intact template made the most contribution to the total frequency of spontaneous mutagenesis. When using the ordered seeding test, the cells were plated on a medium containing an antibiotic at a sublethal dose, which greatly increased the time of one generation (~10 divisions over 14 days) and, as a consequence, the amount of spontaneous damage. In wild-type cells, normally functioning repair systems can effectively do with this relatively small amount of spontaneous damage. However, in cells with faulty repair systems, the amount of spontaneous damage that turn out in the replication fork will be higher compared to the amount in wild-type cells. As a result, the proportion of spontaneous mutations arising in the repair process of lesions will prevail over the proportion of mutations arising from the replication of an intact template. *hat1Δ* mutation troubles the repair process and we observed the growth of spontaneous mutagenesis in the ordered seeding test and a slight change in the level of mutagenesis in the Coulson test. In the ordered seeding test, *hsm3Δ* and *hif1Δ*, mutants showed a much higher frequency of spontaneous mutations than the mutant *hat1Δ*, which epistatized to *hsm3Δ* and *hif1Δ* mutations in the double *hat1Δ hsm3Δ* and *hat1Δ hif1Δ* mutants. Taken together, the results obtained allow us to conclude that the *HSM3* gene product is a new subunit of NuB4 complex.

The inactivation of two anti-recombination helicases Srs2 and Mph1 and Mms4 subunit of endonuclease terminating DNA synthesis in the D-loop suppresses *hsm3*-specific mutagenesis [38,43]. Both of these helicases and Mus81/Mms4 endonuclease decrease the average length of the synthesized DNA region [53,54,55,56,57,58]. Consequently, their inactivation will lead to an increase in the length of the newly synthesized DNA in the D-loop, and this event is the reason for the suppression of *hsm3*- and *hif1*-specific mutagenesis. From this, it follows that the change of polymerases occurs after the destruction of the D-loop and the gap is filled with an erroneous polymerase. We observed the same events in *him1Δ* mutant [44].

Earlier, we have shown that mismatch repair plays a role in *hsm3*-dependent mutagenesis [30]. The mutation frequency in *hsm3Δ pms1Δ* double mutant was significantly higher than in both single mutants. This data shows that mismatch repair substrates arose in the cells of *hsm3Δ* mutant as a result of the attraction of erroneous DNA polymerases. Consistent with this conclusion, we found that Polη inactivation completely blocks *hsm3*-dependent UV mutagenesis. This conclusion is also supported by data showed that Polη-dependent mutagenesis in NBP sites occurs significantly more frequently in *hsm3Δ* mutant than in the double *him1Δ rad30Δ* mutant.

We showed that mutations in *HSM3* gene suppresses UV-induced expression of *RNR* genes.

**Figure 9:**
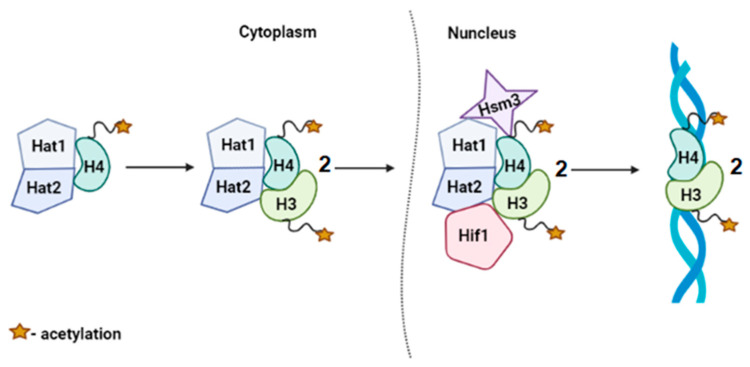
A model describing the potential mechanism of action of the Hsm3p as part of NuB4 complex. This model describes an approximate mechanism of action of Hsm3p, as a subunit of the histone acetyltransferases complex NuB4. Most likely, Hsm3p joins in the Hat1p/Hat2p complex, as well as Hif1p.

Genetic data we obtained are consistent with the results of a study of physical interactions subunits of NuB4 complex with Hsm3p [22,24], indicating that the product of *HSM3* gene can function as part of a NuB4 complex (Figure 9).

## Figures and Tables

**Table 1:** Strains’ genotypes.

Strain	Genotype
11D-LMG-3031	*MATa ade2∆-248 leu2-3.112 ura3-160.188 trp1∆*
5-LMG-3031	*MAT* *a* *ade2* *Δ* *-248 ura3-160,188 leu2-3,112 trp1 hsm3::Kan^R^*
LMG-351	*MATa ade2∆-248 leu2-3.112 ura3-160.188 trp1∆ rad* *1* *::LEU2*
LMG-352	*MAT* *a* *ade2* *∆* *-248 ura3-160,188 leu2-3,112 trp1 rad2::TRP1*
10-CAY-3031	*MAT α ade2∆-248 ura3-160,188 leu2-3,112 trp1 rad52::URA3*
CAY-2	*MAT a ade2∆-248 ura3-160,188 leu2-3,112 trp1 hat1::NAT*
CAY-3	*MAT a ade2∆-248 ura3-160,188 leu2-3,112 trp1 hif1::ura4^+^*(*S. pombe*)
CAY-4	*MAT a ade2∆-248 ura3-160,188 leu2-3,112 trp1 hat1::NAT rad52::URA3*
CAY-6	*MAT a ade2∆-248 ura3-160,188 leu2-3,112 trp1hat1::NAT rad1::LEU2*
CAY-7	*MAT a ade2∆-248 ura3-160,188 leu2-3,112 trp1 hat1::NAT rad2::TRP1*
13-CAY-5-3031	*MAT α ade2∆-248 ura3-160,188 leu2-3,112 trp1 hsm3::Kan ^R^ rad52::URA3*
CAY-9	*MAT α ade2∆-248 ura3-160,188 leu2-3,112 trp1 hif1::ura4^+^(S. pombe) hsm3::Kan ^R^*
CAY-10	*MAT α ade2∆-248 ura3-160,188 leu2-3,112 trp1 hat1::NAT hif1::ura4^+^(S. pombe) hsm3::Kan ^R^*
CAY-11	*MAT α ade2∆-248 ura3-160,188 leu2-3,112 trp1 hif1::ura4^+^(S. pombe) hat1::NAT*
CAY-12	*MAT α ade2∆-248 ura3-160,188 leu2-3,112 trp1 hif1::ura4^+^(S. pombe) rad2::TRP1*
CAY-13	*MAT α ade2∆-248 ura3-160,188 leu2-3,112 trp1 hif1::ura4^+^(S. pombe) rad52::LEU2*
CAY-15	*MAT α ade2∆-248 ura3-160,188 leu2-3,112 trp1 hsm3::Kan ^R^ hat1::NAT*
TAE-151	*MAT α ade2∆-248 ura3-160,188 leu2-3,112 trp1 hsm3::Kan ^R^ sml1::NAT*
TAE-152	*MAT α ade2∆-248 ura3-160,188 leu2-3,112 trp1 rad30::NAT hsm3::Kan ^R^*
TAE-153	*MAT α ade2∆-248 ura3-160,188 leu2-3,112 trp1 rad30::NAT*
TAE-154	*MAT α ade2∆-248 ura3-160,188 leu2-3,112 trp1 hif1::ura4^+^(S. pombe) dun1::NAT*
DVF-15	*MAT α ade2∆-248 ura3-160,188 leu2-3,112 trp1 dun1::NAT hsm3::Kan ^R^*
DVF-16	*MAT α ade2∆-248 ura3-160,188 leu2-3,112 trp1 dun1::NAT*
TAE-155	*MAT a ade2∆-248 ura3-160,188 leu2-3,112 trp1 hsm3-1 hif1::ura4^+^*(*S. pombe*)
6-DVF-3031	*MATα ade2Δ-248 ura3-160,188 leu2-3,112 trp1 sml1::kanMX*

**Table 2:** Rate of spontaneous lethal clones after 1 day of incubation on a dense nutrient medium.

Strains	Rate of Spontaneous Lethal Clones (%)
*WT*	3.8 ± 1.39
*rad52Δ*	10.1 ± 3.15
*hif1Δ*	5.0 ± 1.71
*hif1Δ rad52Δ*	5.3 ± 2.04
*hat1Δ*	11.4 ± 3.97
*hat1Δ rad52Δ*	10.1 ± 1.36

**Table 3:** Spontaneous mutagenesis of resistance to canavanine.

Strain	Ordered Seeding, ×10^−7^	Fluctuation Test, ×10^−7^
*WT*	3.0 ± 0.24	3.0 ± 0.16
*hsm3Δ*	56.6 ± 3.44	3.6 ± 0.89
*hif1Δ*	52.1 ± 5.17	3.9 ± 0.63
*hat1Δ*	27.1 ± 4.12	4.1 ± 0.54
*hat1Δ hif1Δ*	26.9 ± 3.61	4.1 ± 0.33
*hat1Δ hsm3Δ*	23.3 ± 5.70	4.1 ± 0.40

**Table 4:** Types of mutations in the *CAN1* locus after UV irradiation of *hsm3Δ* mutant.

Mutation Type	*n* (%)	f × 10^−5^
Base substitutions	27 (73)	23
Frameshifts	5 (14)	3.4
Tandem double	3 (13)	3.1

f, mutation frequency; *n*, mutation number.

## Data Availability

Not applicable.
